# THE INTERSECTION OF SOCIAL DETERMINANTS OF HEALTH AND FAMILY CARE OF PEOPLE LIVING WITH DEMENTIA

**DOI:** 10.1093/geroni/igad104.0271

**Published:** 2023-12-21

**Authors:** Joseph Gaugler, Soo Borson, Fayron Epps, Regina Shih, Lauren Parker, Lisa McGuire

**Affiliations:** University of Minnesota, Minneapolis, Minnesota, United States; University of Southern California, Los Angeles, California, United States; Emory University, Atlanta, Georgia, United States; RAND Social and Behavioral Policy Program, Santa Monica, California, United States; Johns Hopkins University, Baltimore, Maryland, United States; CDC, Atlanta, Georgia, United States

## Abstract

In this presentation, we will synthesize current research to illustrate the intersection of social determinants of health (SDOHs) and Alzheimer’s disease and related dementia (ADRD) caregiving, and outline how public health can support ADRD family caregivers in the U.S. There is an abundance of research that demonstrates family caregivers of relatives living with ADRD experience adverse health outcomes. In addition, family care for persons with ADRD overlaps with SDOHs. Public health actions such as improved surveillance and identification of ADRD caregivers; building and enhancing community partnerships; advancing dementia-capable health care and related payment incentives; and reducing the stigma of dementia and ADRD caregiving can enhance support and services for families. By engaging in these actions, public health practitioners could more effectively address the myriad of challenges facing ADRD caregivers most at risk for emotional, social, financial, psychological, and health disruption.

